# Case Report: Infusion-Related Reactions to Intravenous Infliximab and Subcutaneous Ustekinumab in Pediatric Crohn's Disease

**DOI:** 10.3389/fped.2021.670703

**Published:** 2021-12-24

**Authors:** Toshihiko Kakiuchi, Sakiko Kimura, Motohiro Esaki, Muneaki Matsuo

**Affiliations:** ^1^Department of Pediatrics, Faculty of Medicine, Saga University, Saga, Japan; ^2^Department of Pharmacy, Saga University Hospital, Saga, Japan; ^3^Division of Gastroenterology, Department of Internal Medicine, Faculty of Medicine, Saga University, Saga, Japan

**Keywords:** allergic reactions, biological agent, infusion-related reactions, L-histidine, infliximab, ustekinumab, pediatrics

## Abstract

**Background:** Although the biological agent ustekinumab (UST) is reported to be effective for Crohn's disease (CD) in pediatric as well as adult patients, data on the efficacy and safety of UST in pediatric patients with CD are limited. Here, we describe the case of a pediatric patient who showed an allergic reaction to UST after subcutaneous (SC) maintenance injections but not immediately after initial intravenous (IV) injection.

**Case Presentation:** A 9-year-old boy presented to our hospital with diarrhea lasting 2 years and weight loss, leading to the diagnosis of CD. After prednisolone (PSL) was tapered and discontinued, he promptly relapsed. According to our institution's protocol, we introduced the biological agent infliximab (IFX) with premedication. Coughing and vomiting was observed after the second dose of IFX and it was changed to adalimumab (ADA). However, the effect of ADA gradually disappeared after 18 months; therefore, it was discontinued and he was treated using UST. The first IV UST dose was given after administering hydrocortisone (HDC), an antiallergic and antipyretic analgesic, as premedication, and no obvious adverse reaction was observed. After 8 weeks, UST was subcutaneously injected without premedication. The patient then complained of nausea, dizziness, and headache within 15 min of UST administration. Therefore, for the third dose of UST, HDC was administered again as premedication. However, nausea, dizziness, and headache presented 10 min after UST administration, resulting in discontinuation of further UST treatment.

**Conclusion:** Careful distinction between “true” infusion-related reactions (IRRs) and anaphylaxis or allergic reactions is necessary to determine whether biological agents can be continued after the development of “so-called” IRRs. For true IRRs, it may be possible to continue using the biological agent with appropriate premedication; however, in cases of anaphylaxis, the biological agent itself should be changed.

## Introduction

Ustekinumab (UST), a humanized monoclonal antibody that binds to the p40 subunit of interleukin (IL)-12 and IL-23, is reportedly effective in treating adult Crohn's disease (CD) (1). However, data on the efficacy and safety of UST in pediatric CD are limited, although it was recently reported to be as effective for pediatric CD as for adult CD (2). The induction dose for pediatric CD is a weight-based intravenous (IV) loading dose followed by subcutaneous (SC) injections of 90 mg UST for maintenance every 8–12 weeks; this is the same dosing used to treat adult CD. To date, there have been some cases of hypersensitivity reactions following IV administration and anaphylaxis following SC administration of UST (3). Thus, the safety of its use should be verified. Here, we describe the case of a pediatric patient who showed an allergic reaction to UST only after the SC maintenance injections but not immediately after the initial IV administration.

## Case Presentation

A 9-year-old boy presented to our hospital with diarrhea since the last 2 years and consequent weight loss. A small bowel series and total colonoscopy (TCS) revealed multiple erosions with a longitudinal tendency from the ileum to the rectum, leading to a diagnosis of CD. The patient was initially treated using 50 mg/kg 5-aminosalicylic acid and 1 mg/kg prednisolone (PSL) and successfully achieved clinical remission. However, he promptly relapsed after PSL dose was tapered and discontinued. We then introduced the biological agent infliximab (IFX; 200 mg) along with antiallergic and antipyretic analgesics administered as premedication as per our institution's protocol. After the second IFX dose 2 weeks later, urticaria (Grade 1) was observed but it disappeared 15 min after stopping treatment (4). Therefore, hydrocortisone (HDC) was administered as a pretreatment before the third IFX dose. However, this time, nausea and cough presented 30 min after IFX administration. The fourth IFX dose was administered at a rate that was three times slower than the initial rate and with the same premedication used at the third IFX dose. However, the patient still presented with coughing and vomiting 15 min after administration. In both series, his symptoms disappeared 30 min after the administration of saline bolus, HDC, and antihistamine infusion (Grade 2). The serum trough level of IFX was <0.10 μg/ml and the anti-IFX antibody titer was 20 times. Despite the adverse reaction, TCS showed that the erosion from the ileum to the rectum had completely disappeared. Adalimumab (ADA; maintenance dose, 40 mg) was used as the next biological agent instead of IFX. The patient showed no adverse reactions to ADA when administered without premedication.

At 1.5 years after the introduction of ADA, the patient's diarrhea and abdominal pain gradually reappeared. Total colonoscopy revealed mucosal redness and multiple small erosions throughout the colorectum. We then swapped ADA with UST as a third biological agent. The first IV does of UST (260 mg) was given after administering HDC (200 mg), an antiallergic and antipyretic analgesic, as premedication, and no obvious adverse reaction was observed. Eight weeks later, second dose of UST (90 mg) was subcutaneously injected without premedication. However, within 15 min, the patient complained of nausea, dizziness, and headache. Therefore, saline bolus, HDC, and antihistamine (Grade 2) were administered, and the symptoms disappeared after 30 min. At the third dose, despite administering HDC as premedication, nausea, dizziness, and headache were observed 10 min after UST administration, compelling the discontinuation of further UST treatment. The adverse symptoms after IFX administration were cough and vomiting, which were different from the nausea, dizziness, and headache symptoms that appeared after UST administration. Both biological agents were administered in the recumbent position because the patient had been diagnosed with unmedicated orthostatic dysregulation prior to the CD diagnosis. No abnormal findings were observed at the injection site after both procedures. Eventually, the biological agent was changed from UST to ADA (maintenance dose 80 mg) and the patient's abdominal symptoms stabilized.

## Discussion

Infusion-related reactions (IRRs) to biological agents are serious safety concerns in the management of inflammatory bowel diseases. However, so-called IRRs include various types of adverse reactions and are thought to have the following possible causes: (1) complement activation-related pseudo-allergy (CARPA), (2) cytokine release syndrome (CRS), (3) immunoglobulin E-mediated anaphylaxis (severe) or allergic reactions (mild–moderate) including cross reactivity, or (4) anaphylactoid reaction (5). The first two lead to “true” IRRs, whereas the latter two are allergic-related reactions to biologic agents.

In the present case, CARPA seems unlikely to have led to the so-called IRR as it frequently occurs at the first administration and decreases after the second administration (6). Although we cannot fully eliminate the possibility that premedication with IV UST prevented CARPA in our patient, adverse reactions occurred at the time of the first and second SC dose of UST, regardless of the application of premedication. Thus, CRS seems less likely to be the cause of the so-called IRR, although the amount of cytokine after IV and SC UST administration was not measured.

Although both immunoglobulin E-mediated anaphylaxis and anaphylactoid reactions cause rapid onset of systemic reactions, anaphylactoid reactions are considered to be generally milder upon repeated administration in contrast to immunoglobulin E-mediated anaphylaxis. Table 1 lists the excipients of IFX, ADA, and UST infusion and the prefilled syringe used for SC injection. The only components not included in ADA but present in both IV and SC UST were UST and L-histidine. The adverse reactions in this case may have been due to sensitization to UST or L-histidine during the first IV dose of UST, which caused allergic reactions upon the first and second SC dose of UST. L-histidine is commonly added as a buffer or stabilizer for UST. The L-histidine in both IV and SC UST may also have played a role in the non-specific release of histamine (7), which could have exacerbated an allergic reaction. Previous studies have assessed the cross-reactivity between antidrug antibodies directed at one biologic agent and those directed at another biologic agent. These studies observed no cross-reactivity between IFX and ADA (8); this could be due to their different immunogenic epitopes. Therefore, it is unlikely that antidrug antibodies directed at a certain biological agent will cross-react with those directed at another biological agent. Based on the above, we speculate that the adverse reactions after IV IFX and SC UST administrations were likely to be allergic reactions.

**Table 1:** Comparison of excipients found in the infliximab, adalimumab, and Ustekinumab infusion and the prefilled syringe used for SC injection.

**Infliximab (vial)**	**Adalimumab** **(syringe and autoinjector)**	**Ustekinumab IV infusion** **(26 ml vial)**	**Ustekinumab SC injection** **(0.5 ml PFS)**
Infliximab 100 mg	Adalimumab 20, 40, and 80 mg	Ustekinumab 130 mg	Ustekinumab 45 mg
Sodium dihydrogen phosphate monohydrate (2.2 mg)			
Disodium hydrogen phosphate dihydrate (6.1 mg)			
		EDTA disodium salt dihydrate (0.52 mg)	
		L-Histidine (20 mg)	L-Histidine (0.5 mg)
		L-Histidine monohydrochloride monohydrate (27 mg)	
		L-Methionine (10.4 mg)	
Polysorbate 80 (0.5 mg)	Polysorbate 80 (0.2, 0.4, 0.8 mg)	Polysorbate 80 (10.4 mg)	Polysorbate 80 (0.02 mg)
Sucrose (500 mg)		Sucrose (2,210 mg)	Sucrose (38 mg)
	D-mannitol (8.4, 16.8, 33.6 mg)		

*SC, subcutaneous; PFS, prefilled syringe; EDTA, ethylenediaminetetraacetic acid*.

In conclusion, careful discrimination between “true” IRRs caused by CARPA or CRS and anaphylaxis or allergic reactions is necessary to determine whether a biologic agent can be continued after the development of so-called IRRs. For biological agents showing true IRRs, it may be possible to continue using them with appropriate premedication; however, for those leading to anaphylaxis, the biological agent itself should be changed.

## Data Availability Statement

The original contributions presented in the study are included in the article/supplementary material, further inquiries can be directed to the corresponding author/s.

## Ethics Statement

Ethical review and approval was not required for the study on human participants in accordance with the local legislation and institutional requirements. Written informed consent to participate in this study was provided by the participants' legal guardian/next of kin.

## Author Contributions

TK was involved in patient care as well as the drafting, review, and revision of the initial manuscript. SK was involved in patient's treatment decision as well as the review and revision of the initial manuscript. ME was involved in patient care as well as the review and revision of the initial manuscript. MM was involved in patient care and project administration, as well as the review and revision of the initial manuscript. All authors approve the final manuscript submission and agree to be accountable for all aspects of the study.

## Conflict of Interest

The authors declare that the research was conducted in the absence of any commercial or financial relationships that could be construed as a potential conflict of interest.

## Publisher's Note

All claims expressed in this article are solely those of the authors and do not necessarily represent those of their affiliated organizations, or those of the publisher, the editors and the reviewers. Any product that may be evaluated in this article, or claim that may be made by its manufacturer, is not guaranteed or endorsed by the publisher.

## Abbreviations

UST: ustekinumab
IL: interleukin
CD: Crohn's disease
IV: intravenous
SC: subcutaneous
TCS: total colonoscopy
PSL: prednisolone
IFX: infliximab
HDC: hydrocortisone
ADA: adalimumab
IRRs: infusion-related reactions
CARPA: complement activation-related pseudo-allergy
CRS: cytokine release syndrome.
